# Mortality in patients with rheumatoid arthritis: a 15-year prospective cohort study

**DOI:** 10.1007/s00296-016-3638-5

**Published:** 2016-12-28

**Authors:** J. van den Hoek, H. C. Boshuizen, L. D. Roorda, G. J. Tijhuis, M. T. Nurmohamed, G. A. M. van den Bos, J. Dekker

**Affiliations:** 1 Amsterdam Rehabilitation Research Center | Reade, PO Box 58271, 1040 HG Amsterdam, The Netherlands; 2 Department of Social Medicine, Academic Medical Center, University of Amsterdam, Amsterdam, The Netherlands; 3 National Institute of Public Health and the Environment, Bilthoven, The Netherlands; 4 Biometrics, Wageningen University and Research Centre, Wageningen, The Netherlands; 5 Amsterdam Rheumatology and Immunology Center, Location Reade, Amsterdam, The Netherlands; 6 Departments of Rehabilitation and Psychiatry, EMGO Institute, VU University Medical Center, Amsterdam, The Netherlands

**Keywords:** Rheumatoid arthritis, Mortality, Cause of death, Comorbidity, Cohort study, Longitudinal studies, Survival

## Abstract

The aim of this study was to investigate (a) the mortality in a clinical cohort of patients with established rheumatoid arthritis in comparison with the general Dutch population over 15 years, (b) the trend in the mortality ratio during the study period, and (c) causes of death and compare these with the general population. In 1997, a sample of 1222 patients was randomly selected from the register of a large rheumatology outpatient clinic. Their mortality and primary causes of death between 1997 and 2012 were obtained from Statistics Netherlands. The standardized mortality ratio (SMR) for all-cause mortality and the number of life-years lost in the study period, adjusted for age, sex, and calendar year, were calculated. A linear poisson regression analysis was performed to evaluate change in all-cause SMR over time. Finally, the SMRs for cause-specific mortality were calculated. The mean age of the population at baseline was 60.4 (SD 15.4) years, and 72.6% of the patients were women. The estimated SMR (95% CI) for all-cause mortality was 1.54 (1.41, 1.67) with about one life-year lost over the study period. There was a trend to decreasing SMR (2% annually, *p* = .07). Mortality was higher compared with the general population for circulatory system diseases, respiratory system diseases, musculoskeletal system diseases, and digestive system diseases (*p* < .05). The observed mortality among patients with RA was 54% higher than in the general population after adjustment for age, sex and calendar year. More than one life-year was lost over 15 years, and the mortality tended to decrease over time. The mortality was higher for cardiovascular, respiratory, musculoskeletal and digestive diseases.

## Introduction

Patients with rheumatoid arthritis (RA) have a higher mortality risk than the general population. Mortality rates in persons with RA are around 1.5 times higher than in the general population, with similar patterns over the last 50 years [1].

Causes of death that are increased in comparison with the general population are cardiovascular diseases, respiratory diseases and infections. The higher mortality rate is particularly caused by cardiovascular (CV) disease [2–7], but only partly caused by the higher prevalence of cardiovascular risk factors [8, 9]. The additional risk depends on systemic inflammation [10]. Other increased causes of death are respiratory diseases and infections [6, 7, 11] most often due to respiratory infection/pneumonia [4–6]. The increased infection risk may be attributed to the impaired immune function in RA or an effect of immunosuppressive therapy. In the most recent studies investigating cause-specific mortality, only a limited number of causes of death were studied [12], the number of patients who died during follow-up was small, and most data came from studies conducted before 2004 [11, 13]. There is thus a need to study the cause-specific mortality for a wide range of causes, in a large cohort, using more recent mortality data.

The treatment of patients with RA has been improved over time. The focus is now on tight disease control with much earlier initiation of intensive treatment [14]. From the 1990s, high-dose treatment with disease-modifying anti-rheumatic drugs (DMARDs), particularly methotrexate started and biologicals were applied from 2000 onwards. Meta-analyses suggest that DMARDs (particularly methotrexate) reduce CV risk [15] and there is accumulating observational evidence that this also applies for biologicals, particularly the TNF blockers [16, 17].

Although the management of RA has improved, outcomes about mortality rates of observational studies that started around 2000 differ. Some studies report that the mortality in patients with RA was similar to that of the general population [18, 19], while other studies showed that the mortality in patients with RA was higher [20, 21] or that the mortality gap with the general population was even increasing [11]. Reason for the different results can be the changing RA treatment during the last two decades as described above [14] or to the different types of cohorts and follow-up time [22]. Given these conflicting results, there is thus also an obvious need to evaluate the risk of mortality in a large sample of patients with RA, over a long period, using more recent mortality data. The need to study the mortality in long-term clinical cohorts of RA patients was underscored by a recent editorial [23].

Hence, the objectives of this study were (a) to investigate the mortality in a clinical cohort of patients with established RA and to compare this with the general Dutch population, (b) to assess the trend in the mortality ratio during the study period, and (c) to examine the causes of death.

## Patients and methods

### Study design and population

In 1997, our research group started a cohort study on comorbidity [24] and the impact on health outcomes and mortality [25–27] in patients with RA. A sample of 1222 patients was randomly selected from the register of a large rheumatology outpatient clinic in Amsterdam, which included the patients from seven allied outpatient clinics. For inclusion in the study, patients had to fulfil the following eligibility criteria: (a) be diagnosed with RA according to the American College of Rheumatology (ACR) Criteria for RA [28], (b) be 16 years of age or older, and (c) have visited a rheumatologist at least once in the previous 2 years.

### Assessments

All 1222 patients were linked to Statistics Netherlands’ mortality records for the period 1997–2012 [29, 30]. The linkage of the patients to the mortality data was anonymous, using only date of birth, sex, and, if available, civil registration number, and was performed by staff of Statistics Netherlands [31]. Its databank provides death/life status, death dates, and primary and secondary causes of death. For this study, we used primary causes of death only. Causes of death were classified according to the Internal Classification of Diseases system (ICD-10) of the World Health Organization [32]. For patients who could not be linked to these records, data were obtained from the outpatient clinic register. In order to compare patients with the general population, age and sex specific mortality data and causes of death for the general population were provided by Statistics Netherlands [30].

### Statistical analyses

The standardized mortality ratio (SMR) was computed in order to compare the mortality in the RA cohort with the general population. The SMR was calculated as the ratio of the number of observed deaths in a study population divided by the number of expected deaths if the study population had the same age, sex, and calendar year specific rates as the general population. To calculate the expected death rates, we used the mortality rates of all Dutch inhabitants. So, in calculating the expected number of deaths there was adjusted for age, sex, and calendar year. We calculated the SMR for all-cause mortality, for each year, the mean of 3 years, the mean of 5 years, and the mean of all 15 years.

The number of life-years lost was calculated as follows. First, the partial life expectancy for the RA cohort was calculated from the start of the study to 15 years later. This was also done for the general Dutch population, using a sex- and age-matched cohort. Second, we calculated the difference in the expected life-years during the study period between the RA cohort and the general population.

To investigate if there was a trend towards an increase or decrease in the SMR over time, a poisson regression analysis was performed with log link function. Because there are only a few points in time, a simple model was used. Both identity link function and log link function showed a good fit when checking the deviance of the residuals [33].

Because the exact date of inclusion in the cohort was not clear, and as this might bias the calculated SMR for this year, the 1997 data were excluded from this analysis.

Finally, the SMR for cause-specific mortality was calculated. Because of limited numbers, we calculated the mean SMR for all 15 years.

## Results

### Study population

Of the 1222 patients who were selected at baseline, 1208 patients (99%) could be linked to Statistics Netherlands. Of the 14 patients who could not be linked, five died, according to the outpatient clinic’s register. For these five patients, no information about cause-specific mortality was available. No information about mortality could be obtained for nine patients. These nine patients were excluded from the analyses. Therefore, data of 1213 patients were used for our analyses. The mean age of these patients at baseline was 60.4 (SD 15.4) years, and 72.6% of the patients were women. Data on disease duration were available for a sub-cohort of patients (*n* = 882). The mean disease duration in this sub-cohort was 5.0 (IQR 2.0–14.0) years [18]. A total number of 540 patients died during the study period. The relative number of patients that died during the study period was 45% (540/1213). We expected based on the mortality rates of the general population that 29% (352/1213) would have died during the study period (Table 1).

**Table 1:** Number of observed deaths for each year of the study period

Year	*N*	Total	Percentage
1997	19	1213	1.57
1998	41	1194	3.43
1999	43	1151	3.74
2000	38	1113	3.41
2001	43	1070	4.02
2002	45	1025	4.39
2003	35	990	3.54
2004	38	952	3.99
2005	42	910	4.62
2006	37	873	4.24
2007	35	838	4.18
2008	21	817	2.57
2009	38	779	4.88
2010	21	758	2.77
2011	21	737	2.85
2012	23	714	3.22

### All-cause mortality

The estimated SMR (95% confidence interval) for all-cause mortality was 1.54 (1.41, 1.67) over the period of 15 years. This indicates a 54% higher risk of mortality compared with the general Dutch population. In females, it was 1.62 (1.46, 1.80), and in males, it was 1.32 (1.13, 1.55).

The partial life expectancy for the Dutch population for the same period of 15 years was 13.4 years. The corresponding life expectancy for the RA cohort was 12.2 years. The number of expected life-years the RA cohort lost compared with the general population during the study period was 1.2 years.

### Change in all-cause mortality

**Fig. 1:**
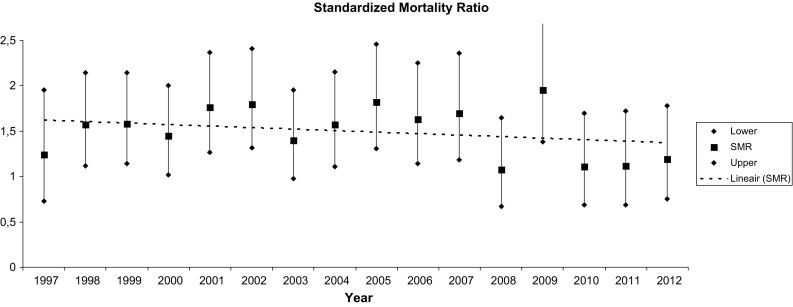
Annual standardized mortality ratio and 95% confidence interval

Figure 1 shows the annual SMR. Table 2 shows the mean SMR for all-cause mortality over 3 years, over 5 years, and over the total study period. The outcome of the regression analysis showed that there was a trend to decreasing SMR (2% annually, *p* = .07).

**Table 2:** All-cause mortality in RA population

		Confidence interval			Confidence interval		Total	Confidence interval	
	Men	Lower	Upper	Women	Lower	Upper		Lower	Upper
(a) SMR (mean 3 years)									
1997–2000	1.42	0.72	2.50	1.63	1.07	2.37	1.54	1.08	2.14
2001–2003	1.29	0.64	2.31	1.80	1.21	2.57	1.65	1.19	2.24
2004–2006	1.57	0.82	2.71	1.75	1.14	2.57	1.68	1.20	2.30
2007–2009	1.45	0.69	2.70	2.07	1.36	3.01	1.58	1.07	2.24
2010–2012	0.76	0.25	1.82	1.56	0.96	2.42	1.14	0.71	1.73
(b) SMR (mean 5 years)									
1997–2002	1.49	0.78	2.58	1.66	1.07	2.44	1.63	1.15	2.22
2003–2007	1.36	0.67	2.46	1.75	1.15	2.55	1.63	1.15	2.24
2008–2012	1.02	0.39	2.17	1.42	0.85	2.24	1.29	0.83	1.91
(c) SMR (mean 15 years)									
1997–2012	1.32	1.13	1.55	1.62	1.46	1.80	1.54	1.41	1.67

*SMR* standardized mortality ratio, adjusted for age and gender

### Cause-specific mortality

The most frequent causes of death for the RA cohort were circulatory system diseases (32%), neoplasms (21%), and respiratory system diseases (12%). Table 3 shows the SMR for all causes of death compared with the general Dutch population. The RA cohort mortality was higher for circulatory system diseases, respiratory system diseases, musculoskeletal system diseases, and digestive system diseases (*p* < .05).

**Table 3:** Cause-specific mortality in RA population (*N* = 535) over 15 years (1997–2012)

	Observed	Expected	SMR	Confidence interval	
				Lower	Upper
Circulatory system diseases	172	140	1.23	1.05	1.43
Neoplasms	112	108	1.03	0.85	1.24
Respiratory system diseases	62	44	1.42	1.09	1.82
Musculoskeletal and connective tissue diseases	49	3	17.4	12.85	22.97
Digestive system diseases	29	16	1.79	1.20	2.57
Mental and behavioural disorders	23	22	1.04	0.66	1.57
Abnormal clinical and laboratory signs not elsewhere classified	20	18	1.09	0.66	1.68
External causes of morbidity and mortality	17	11	1.55	0.90	2.48
Endocrine, nutritial, and metabolic diseases	14	14	1.01	0.55	1.69
Infections	10	5	1.90	0.91	3.50
Nervous system diseases	10	12	0.86	0.41	1.57
Genitourinary system, skin, subcutaneous tissue, blood, and blood-forming organs diseases	17	13	1.29	0.75	2.06

*SMR* standardized mortality ratio, adjusted for age and gender

## Discussion

This 15-year study showed that the mortality in patients with RA is still higher than that of the general population, but seems to decrease over time. Mortality was higher for circulatory, respiratory, musculoskeletal, and digestive system diseases.

Patients with RA had a 54% higher risk of mortality compared with the general population. This shows that excess mortality in RA still occurs and that the overall mortality rate is still as high as it was in the past decades [1, 34].

The number of life-years lost during the 15-year study period compared with the general population was about 1 year. The mean age at baseline was 60 years, and the 15 year follow-up period is, roughly, half the period needed to observe the full life expectancy. Under this assumption, this results in the estimated total number of life-years lost being of 2 years for a 60-year-old patient.

Likewise, the mortality ratio tended to decrease over time. This is underscored by the fact that the mortality ratio in the last 3 years of the study period was lower than in the preceding years and comparable to that of the general population. This trend towards a decrease in mortality might reflect the introduction of more intensive treatment in the last two decades. Other explanations for the decreasing mortality in this study might be that this cohort that was studied was a fixed cohort, hence new patients could not enter the study. As a result, the improved mortality could be a survival effect, in which patients with more “severe” RA died at the start of the study period (for example patients with shorter disease duration or with rheumatoid factor positive) and less “severe” patients survived and were followed up to the end of the study, which resulted in lower SMR at the end of the study period. We were able to study the association between disease duration and mortality (adjusted for age and gender) in a sub-cohort of 882 patients. There was no association between disease duration and mortality, which makes it more plausible that adjusting for disease duration would not have changed our results (data not shown).

Cause-specific mortality was determined through the primary cause of death. The primary cause of death is defined as the underlying cause of death, which started a sequence that finally resulted in death. This study showed that musculoskeletal and connective tissue diseases were the primary cause of a high number of deaths. Our results differ from other studies, which reported that RA is the primary cause of death in only a few cases [38]. This might be due to the definition of primary cause of death used by Statistics Netherlands. In their databank, the primary cause of death is defined as the underlying cause of death or the disease that started a sequence of incidents that resulted in death [39]. The primary cause of death in the category musculoskeletal and connective tissue diseases was mostly RA and other types of arthritis. The secondary cause of death in patients who were classified as having a musculoskeletal and connective tissue disease as primary cause of death was injury, poisoning, and certain other consequences of external causes in 20%, circulatory diseases in 16%, respiratory diseases in 16%, and infections in 12% of the cases (data not shown). Different definitions of cause of death, used in national registries, will have important implications for study results. Therefore, results addressing cause-specific mortality, originating from different national registries, should be interpreted with caution.

Causes of death that were higher compared with the general population were diseases of the circulatory, respiratory, musculoskeletal, and digestive system. The change in health care could have resulted in different causes of death.

It is expected that current tight disease control translates into a lower CV mortality. Results of this study showed that the majority of the patients died due to CV diseases, which appeared to be the primary cause of death in 32% of the cases. This is somewhat lower than reported in older studies (conducted before implementation of intensive treatment and tight control of disease) where 40% of the patients with RA died due to CV diseases [34]. Our lower CV death rate might be related to currently applied medications. Nevertheless, it is still significantly higher than in the general population.

Mortality due to respiratory disease was also higher in the RA cohort compared with the general population. Although we did not look in detail to which specific respiratory diseases were registered in our cohort, other literature suggests that in most cases this will be respiratory infection [5]. The use of biologicals is associated with more severe infections [35–37], and therefore, it is expected that mortality due to respiratory disease would be higher than in the general population.

In our study, infection as a cause of death was not higher than that of the general population, while other studies report a higher incidence [34]. Infection was, however, often the secondary cause of death when RA was the primary cause of death. Adding the number of patients with infection as a secondary cause of death to the number of patients with infection as a primary cause of death resulted in a significantly higher cause-specific mortality ratio for infection (data not shown).

This is the first Dutch study reporting SMRs until 2012 and the first Dutch study reporting the number of life-years lost in a well-defined cohort of patients with established RA. Moreover, the high number of deaths made it possible to study the cause-specific SMRs for a wide range of causes of death. Another strength of this study was that 99% of the patients could be linked to the Municipal Register of the Statistics Netherlands. Furthermore, this study was conducted in a period in which the treatment of patients with RA changed (focus on tight disease control with the use of high-dose DMARDS from the 1990s and biologicals from 2000), which made it possible to investigate the effect of the new medication. However, a limitation of the study was that no comparisons could be made between the mortality in the study population and the general population corrected for confounders, such as use of medication, smoking, autoantibody profile, and structural damage, because data addressing all these confounders were not available.

In this study, the mortality rate was calculated using indirect standardization. An advantage of indirect standardization is that only information about the total number of deaths for each year in the study population is needed. Thus, the standardized mortality rate can be calculated even if age-specific death rates of the study population at issue are not available or if the number of deaths for each age group is small [40]. A disadvantage is that it represents mortality for an age and gender distribution similar to the study population at issue. By comparing SMRs between studies, it is important to keep in mind that differences in SMRs can be caused by differences in age or gender distribution between study populations at issue.

Comparing the mortality in our RA cohort with the mortality in the general Dutch population may result in selection bias if the RA cohort does not represent the national patient population because of regional differences. However, all patients in this study were recruited from outpatient clinics which have patients referred both from Amsterdam and the northwest part of the Netherlands. Although the mortality in Amsterdam is higher than the mortality in the general Dutch population, the mortality in Amsterdam combined with the northwest part of the Netherlands is comparable to the general Dutch population [41]. For future research, however, we recommend to study the mortality in a RA cohort origination from a national registry.

### Clinical relevance

In patients with established RA, the mortality risk is still higher than the mortality in the general Dutch population, with more than a life-year lost over a period of 15 years. As the primary cause of death is mostly cardiovascular disease, screening for and providing information about cardiovascular diseases (and cardiovascular risk factors) in the rheumatology practice are important. On the other hand, the decrease in mortality coincided with changes in medication policy. Hence, tight disease control is also important to lower the high mortality risk in RA.
